# A Temperature-Sensitive Recombinant of Avian Coronavirus Infectious Bronchitis Virus Provides Complete Protection against Homologous Challenge

**DOI:** 10.1128/jvi.01100-22

**Published:** 2022-08-16

**Authors:** Sarah Keep, Phoebe Stevenson-Leggett, Giulia Dowgier, Katalin Foldes, Isobel Webb, Albert Fones, Kieran Littolff, Holly Everest, Paul Britton, Erica Bickerton

**Affiliations:** a The Pirbright Institute, Surrey, United Kingdom; Loyola University Chicago

**Keywords:** avian, coronavirus, infectious bronchitis virus, temperature sensitivity, vaccine

## Abstract

Avian coronavirus infectious bronchitis virus (IBV) is the etiological agent of infectious bronchitis, an acute highly contagious economically relevant respiratory disease of poultry. Vaccination is used to control IBV infections, with live-attenuated vaccines generated via serial passage of a virulent field isolate through embryonated hens’ eggs. A fine balance must be achieved between attenuation and the retention of immunogenicity. The exact molecular mechanism of attenuation is unknown, and vaccines produced in this manner present a risk of reversion to virulence as few consensus level changes are acquired. Our previous research resulted in the generation of a recombinant IBV (rIBV) known as M41-R, based on a pathogenic strain M41-CK. M41-R was attenuated *in vivo* by two amino acid changes, Nsp10-Pro85Leu and Nsp14-Val393Leu; however, the mechanism of attenuation was not determined. Pro85 and Val393 were found to be conserved among not only IBV strains but members of the wider coronavirus family. This study demonstrates that the same changes are associated with a temperature-sensitive (*ts*) replication phenotype at 41°C *in vitro*, suggesting that the two phenotypes may be linked. Vaccination of specific-pathogen-free chickens with M41-R induced 100% protection against clinical disease, tracheal ciliary damage, and challenge virus replication following homologous challenge with virulent M41-CK. Temperature sensitivity has been used to rationally attenuate other viral pathogens, including influenza, and the identification of amino acid changes that impart both a *ts* and an attenuated phenotype may therefore offer an avenue for future coronavirus vaccine development.

**IMPORTANCE** Infectious bronchitis virus is a pathogen of economic and welfare concern for the global poultry industry. Live-attenuated vaccines against are generated by serial passage of a virulent isolate in embryonated eggs until attenuation is achieved. The exact mechanisms of attenuation are unknown, and vaccines produced have a risk of reversion to virulence. Reverse genetics provides a method to generate vaccines that are rationally attenuated and are more stable with respect to back selection due to their clonal origin. Genetic populations resulting from molecular clones are more homogeneous and lack the presence of parental pathogenic viruses, which generation by multiple passage does not. In this study, we identified two amino acids that impart a temperature-sensitive replication phenotype. Immunogenicity is retained and vaccination results in 100% protection against homologous challenge. Temperature sensitivity, used for the development of vaccines against other viruses, presents a method for the development of coronavirus vaccines.

## INTRODUCTION

The *Gammacoronavirus* infectious bronchitis virus (IBV), first identified in 1937, is a globally economically important pathogen of domestic fowl (1). Infection results in the predominantly respiratory disease infectious bronchitis (IB), with birds classically presenting with watery eyes, nasal discharge, snicking, tracheal rales, and lethargy (2). In addition, IBV-infected birds exhibit reduced tracheal cilium movement and/or ciliostasis (the complete cessation of ciliary activity); this renders the birds susceptible to secondary bacterial infections that can lead to mortality (3, 4). Although IBV primarily replicates in the epithelial cells of the upper respiratory tract, it is also able to infect epithelial cells of the kidney, enteric tract, and oviducts, which can lead to more severe clinical disease, including nephritis (1, 5). IBV is therefore not just a welfare concern but also of major concern to global poultry industries due to its impact on weight gain and egg production, in terms of both decreases in the number and quality produced. Poultry remains an important global food source, and IBV is therefore a major consideration for global food security.

There are several IBV strains and variants, categorized by both serotype and genotype (6), of which many cocirculate. Each geographical region has strains and/or variants of concern, and some are of global concern, including M41, a Massachusetts serotype, and the GI-1 genotype, a strain of IBV (5, 6). IBV is currently controlled through vaccination with a mixture of both live-attenuated and inactivated vaccines (7). Vaccination programs are tailored to specific geographical regions since vaccines typically offer limited cross protection between strains (1, 5). Live-attenuated vaccines are generated through serial passage of a virulent field isolate in embryonated hens’ eggs; attenuation typically requires more than 80 passages (8, 9). Young chicks are vaccinated with live-attenuated vaccines that are administered *en masse* through sprays or via drinking water. Breeders and layers are then boosted at defined intervals with either live attenuated or inactivated vaccines (5). Live-attenuated vaccines are favored due to the ease of application; however, there is a risk of vaccine breakdown and reversion to virulence. The latter is compounded since the exact molecular mechanism of attenuation is unknown, and research has indicated that only a few consensus-level mutations are acquired over the passaging process, providing a short route back to virulence (10).

There is a drive to develop rationally attenuated vaccines. Past research has investigated vaccines consisting of the spike (S) glycoprotein delivered as DNA or by viral vectors (11–17). The S glycoprotein is the major attachment protein and mediates virus entry. It is the main target for vaccine development since several studies have demonstrated it induces virus-neutralizing antibodies (18–21). Vaccines currently employed against the human pandemic severe acute respiratory syndrome coronavirus 2 (SARS-CoV-2) share this methodology, aiming to induce neutralizing antibodies against S (22–24). Although these vaccines offer a good degree of protection against severe disease, especially after multiple vaccinations, they do not offer complete protection in terms of mild to moderate clinical disease or SARS-CoV-2 infection and replication. Interestingly, the protection induced by vaccines that are based solely on S for the control of IBV was not considered high enough for use against IBV in the field (5, 11–17). A next generation of rationally attenuated IBV vaccines is therefore required, whereby the mechanism of attenuation is known, and the degree of attenuation is also known, so that different vaccines do not have differing ranges of efficacy. In addition, such vaccines are more stable with respect to back selection due to their clonal origin. The genetic populations of the vaccines are more homogeneous and, more importantly, lack the presence of parental pathogenic viruses; the potential for back selection is therefore removed.

Our previous research has centered on using the attenuated recombinant IBV (rIBV), Beau-R, a molecular clone of the highly attenuated Beaudette-CK strain (25) to express heterologous S genes (17, 26, 27). Using a rIBV as a vaccine backbone has its advantages since vaccination mimics the route of natural infection, thereby stimulating both local and systemic immune responses. Beau-R expressing the S gene from the pathogenic field strain 4/91(UK) induces a partial protection of ~65%, as defined by tracheal ciliary activity, against homologous challenge (26, 27). Research has highlighted that the replication of rIBV Beau-R *in vivo* is highly restricted and *in vitro* is completely inhibited at 41°C, the core body temperature of a chicken (28). We hypothesized that while the replication of the Beau-R backbone is perhaps too sensitive to temperature, and thus too restricted *in vivo* to induce full protection, that temperature sensitivity could be used to generate live-attenuated vaccine viruses (28). Temperature sensitivity is not a novel topic in the field of virology and has been used extensively for research into vaccines against influenza viruses (29).

Our previous work identified that the sequence of M41-R, although based on the pathogenic M41-CK strain, had several nucleotide differences, including four nonsynonymous changes located in nonstructural proteins (Nsps) 10, 14, 15, and 16 that resulted in the amino acid changes Pro85Leu, Val393Leu, Leu183IIle, and Val209Ile, respectively (30). M41-R had no observable replication defects *in vitro* but was identified to be attenuated both *in ovo* and *in vivo*. The amino acids identified in Nsps 10 and 14 within M41-R, Leu85 and Leu393, respectively, were essential for the attenuated phenotype. In this study, we investigated whether temperature sensitivity at 41°C was a potential mechanism responsible for the attenuated phenotype associated with M41-R. We identified that the replication of M41-R *in vitro* is sensitive to temperature at 41°C compared to M41 CK and showed that two of the nonsynonymous changes that differentiate M41-CK from M41-R, located within Nsps 10 and 14, resulted in the temperature-sensitive (*ts*) replication phenotype. We also demonstrated that RNA replication of M41-R is reduced at 41°C compared to replication at 37°C. However, M41-R, although attenuated *in vivo*, is able to replicate to a higher degree at 41°C in comparison to the highly attenuated rIBV Beau-R. Finally, we demonstrated that vaccination of chickens with M41-R induces 100% protection, as defined by the standards set by the *European Pharmacopeia* (2020), against homologous challenge with M41-CK, thereby demonstrating that temperature sensitivity can be used in the development of novel and innovative live-attenuated IBV vaccines.

## RESULTS

### The *in vitro* replication of M41-R is sensitive to temperature.

Previous research demonstrated that the replication of the attenuated rIBV M41-R both *in vitro* and in *ex vivo* tracheal organ cultures (TOCs) was largely comparable to the parental pathogenic virus M41-CK, as well as the pathogenic rIBV M41-K (30). The rIBV M41-K was generated through modification of the M41-R genome to replace the nucleotide residues at positions 12137, 18114, 19047 and 20139 in Nsps 10, 14, 15, and 16, respectively, to match the pathogenic M41-CK sequence (30). The changes made with the M41-R backbone were as follows: U12137C in Nsp 10, C18114G in Nsp 14, A19047U in Nsp 15, and A20139G in Nsp 16. This consequently resulted in the amino acid changes, Leu85Pro, Leu393Val, Ile183Leu, and Ile209Val in Nsps 10, 14, 15, and 16, respectively, where the number stated specifies the amino acid residue in the corresponding Nsp.

**FIG 1:**
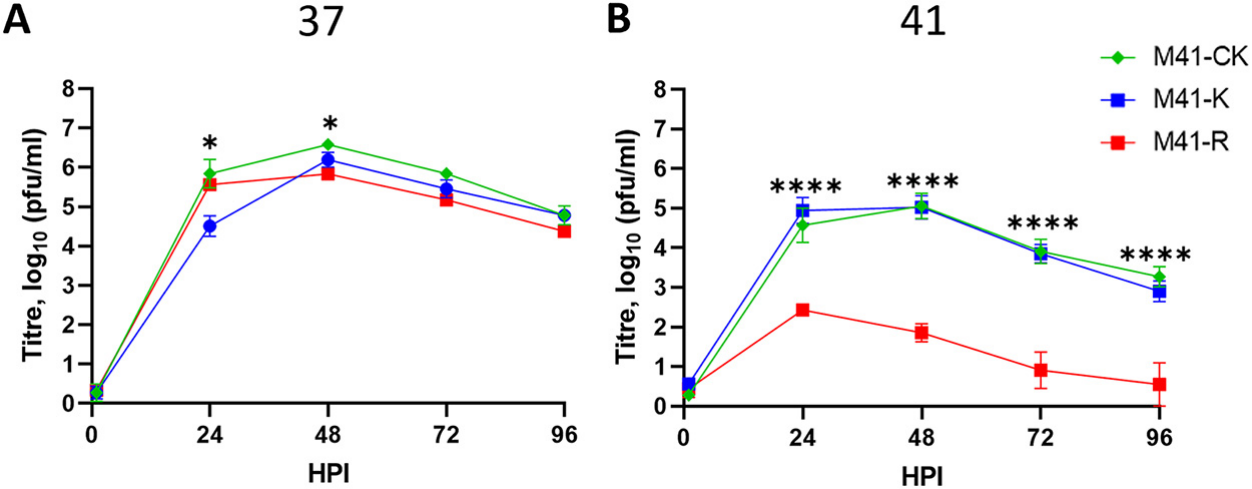
*In vitro* replication of M41-R is reduced at 41°C. CK cells were inoculated with 10^4^ PFU (MOI ~ 0.01) of either M41-CK, M41-K, or M41-R and incubated at either 37°C (A) or 41°C (B). The quantity of infectious progeny in the supernatant harvested at 24-h intervals was determined via titration in triplicate on CK cells. Each point represents the average (mean) of three independent experiments, with error bars indicating the standard errors of the mean (SEM). At each time point a one-way ANOVA was used to assess statistical differences with a Tukey test for multiple comparisons. Differences are indicated by asterisks between M41-CK and M41-K at 24 h and between M41-CK and M41-R at 48 h (*, *P* < 0.05) and between both M41-CK and M41-K in comparison to M41-R (****, *P* < 0.0001; no statistical difference was identified between M41-CK and M41-K).

In our previous research, all growth kinetic assays were completed at 37°C (30), a temperature relatable to the upper respiratory tract, including the nasal turbinates as well as the upper sections of the trachea (28, 31). To investigate whether M41-R replication *in vitro* was affected by increased temperature, a growth kinetic assay was carried out at 41°C, the core body temperature of a chicken and therefore the temperature of the lower sections of the trachea, as well as the lower respiratory tract, including the lungs (31). Primary CK cells were inoculated with either M41-CK, M41-K, or M41-R and incubated at either 37 or 41°C for 96 h postinfection (hpi). The resulting supernatants were titrated at various time points to establish the quantity of infectious progeny virus, thereby indicating the level of productive replication (Fig. 1). The titers were largely comparable between the three viruses at 37°C, with the exception that titers of M41-K were slightly lower than both M41-R and M41-CK at 24 hpi (Fig. 1A). However, the titers of M41-R were observed to be significantly lower than both M41-K and M41-CK at all the time points assessed at 41°C, indicating a decrease in productive replication (*P* < 0.05; Fig. 1B). There were no differences between the growth of M41-K and M41-CK at the two temperatures. These results indicate that the higher temperature of 41°C negatively affects the productive replication of M41-R *in vitro*. The only difference in genomic sequence between M41-K and M41-R are those nucleotide residues that were modified between the two virus genomes, implying that the four amino acid differences in Nsp 10, 14, 15, and 16 are involved in the *ts* phenotype observed for M41-R. This therefore suggests that the presence of one or more of the amino acids—Leu85 in Nsp 10, Leu393 in Nsp 14, Ile183 in Nsp 15, or Ile209 in Nsp 16—confers a *ts* replication phenotype to M41-R.

### Leu85 in Nsp 10 with leu393 in Nsp 14 results in the M41-R *ts* phenotype.

Previous research identified the amino acid differences between M41-K and M41-R responsible for the restoration of pathogenicity (30). The M41-R backbone was modified in order to generate a series of rIBVs containing different combinations of the identified nucleotides at positions 12137, 18114, 19047, and 20139 in Nsps 10, 14, 15, and 16 that resulted in nonsynonymous amino acid differences (Table 1). The *in vitro* growth kinetics for each rIBV were comparable to M41-K at 37°C (30). However, *in vivo* analysis of these rIBVs showed that the replacement of T12137C (Leu85Pro) in Nsp 10 and of C18114G (Leu393Val) in Nsp 14, within the M41-R backbone, conferred a pathogenic *in vivo* phenotype (30).

**TABLE 1:** Summary of rIBVs indicating the amino acids present in Nsps 10, 14, 15, and 16 and their associated *in vivo* phenotypes^*a*^

IBV		Amino acid(s) associated with Nsps 10, 14, 15, and 16	*In vivo* phenotype
New name	Previous name		
M41-CK	M41-CK	Nsp10-P85, Nsp14-V393, Nsp15-L183, Nsp16-V209	Pathogenic
**M41-R**	M41-R	**Nsp10-L85**, **Nsp14-L393**, Nsp15-I183, Nsp16-I209	**Attenuated**
M41-K	M41-K (M41-R_Nsp10-L85-Nsp14-L393-Nsp15-I183-Nsp16-I209_)	Nsp10-P85, Nsp14-V393, Nsp15-L183, Nsp16-V209	Pathogenic
**M41K_Nsp14-L393-Nsp15-I183-Nsp16-I209_**	M41R-nsp10rep	Nsp10-P85, **Nsp14-L393**, Nsp15-I183, Nsp16-I209	**Attenuated**
M41-K_Nsp15-I183-Nsp16-I209_	M41R-nsp10.14rep	Nsp10-P85, Nsp14-V393, Nsp15-I183, Nsp16-I209	Pathogenic
**M41-K_Nsp14-L393 Nsp16-I209-_**	M41R-nsp10.15rep	Nsp10-P85, **Nsp14-L393**, Nsp15-L183, Nsp16-I209	**Attenuated**
**M41-K_Nsp14-L393-Nsp15-I183_**	M41R-nsp10.16rep	Nsp10-P85, **Nsp14-L393**, Nsp15-I183, Nsp16-V209	**Attenuated**
M41-K_Nsp16-I209_	M41R-nsp10.14.15rep	Nsp10-P85, Nsp14-V393, Nsp15-L183, Nsp16-I209	Medium
M41-K_Nsp15-I183_	M41R-nsp10.14.16rep	Nsp10-P85, Nsp14-V393, Nsp15-I183, Nsp16-V209	Pathogenic
**M41-K_Nsp14-L393_**	M41R-nsp10.15.16rep	Nsp10-P85, **Nsp14-L393**, Nsp15-L183, Nsp16-V209	**Attenuated**
**M41-K_Nsp10-L85_**	M41R-nsp14.15.16rep	**Nsp10-L85**, Nsp14-V393, Nsp15-L183, Nsp16-V209	**Attenuated**

a All of the rIBVs are based on M41-R, as reported by Keep et al. (30), in which various combinations of the four amino acids in Nsps 10, 14, 15, and 16 were replaced in M41-R, with those identified in pathogenic M41-CK. The rIBV M41-K represents a virus with all four amino acids replaced in M41-R (30). The second column indicates the old name as reported previously. For clarity, we have renamed the rIBVs based on the rIBV M41-K, the pathogenic molecular clone of M41-R. The new names indicate the M41-R amino acid within the corresponding M41-K Nsp, and the third column denotes the four amino acids present in four Nsps 10, 14, 15, and 16 for each rIBV. Viruses that are attenuated are indicated in boldface, and the amino acid responsible for attenuation in either Nsp 10 or Nsp 14 are also in boldface. Pathogenicity was determined based on *in vivo* ciliary activity data (30). An attenuated phenotype is defined as an average ciliary activity of >50% on days 4 and 6 postinfection (p.i.), a pathogenic phenotype is defined as an average ciliary activity of <50% on days 4 and 6 p.i., and a medium phenotype is defined as an average ciliary activity of <50% on either day 4 or 6 p.i.

**FIG 2:**
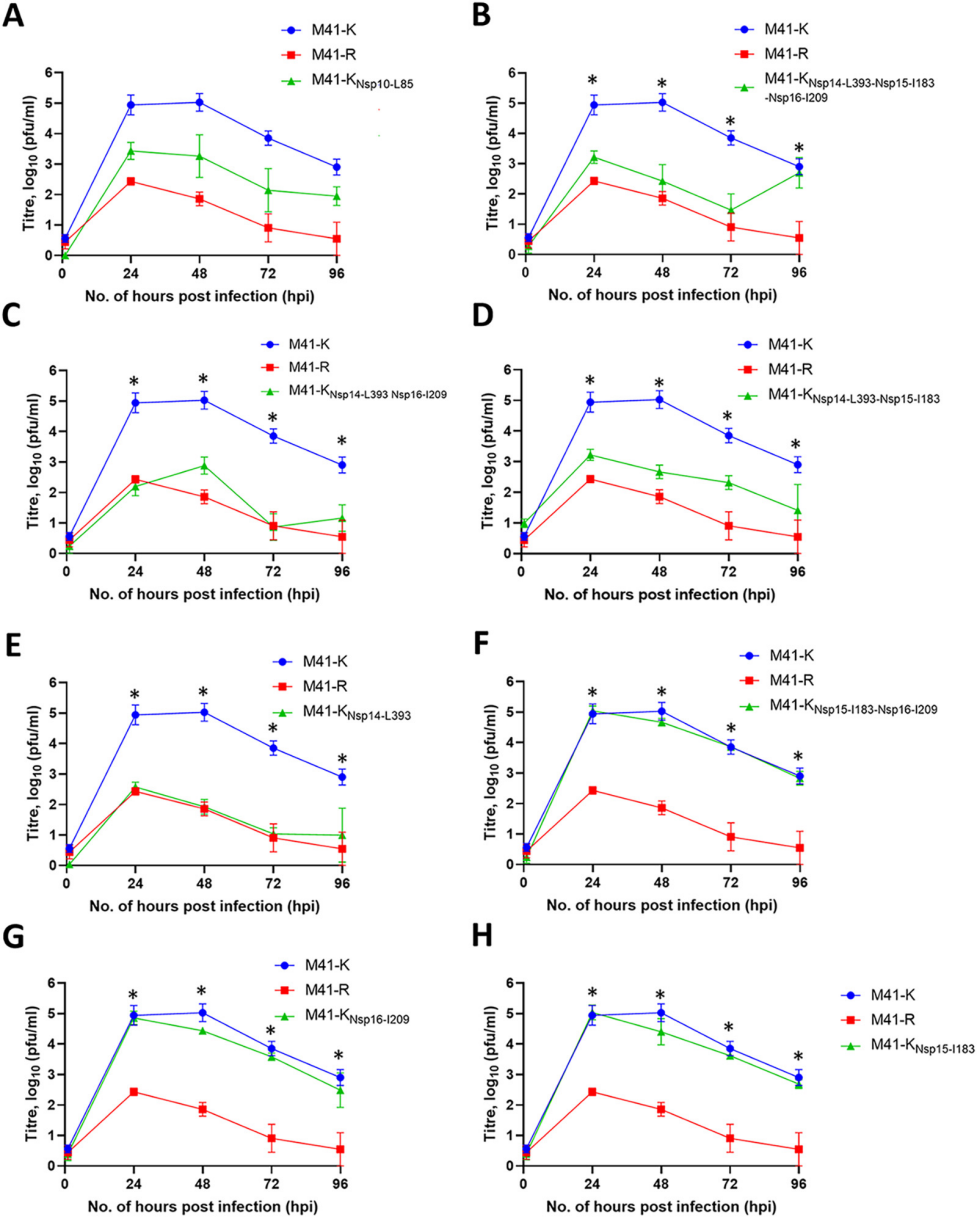
The amino acid changes Leu85Pro in Nsp 10 and Leu393Val in Nsp 14 confer the ability of modified M41-R to replicate at 41°C. Primary CK cells were inoculated with 10^4^ PFU (MOI ~ 0.01) of M41-K and M41-R alongside the test rIBV (A) M41-K_nsp10-L85_, (B) M41-K_nsp14-L393-Nsp15-I183-Nsp16-I209_, (C) M41-K_nsp14-L393-Nsp16-I209_, (D) M41-K_nsp14-L393-Nsp15-I183_, (E) M41-K_Nsp14-L393_, (F) M41K_nsp15-I183-Nsp16-I209_ (G) M41-K_nsp16-L209_ or (H) M41-K_nsp15I183_. The quantity of infectious progeny in supernatant harvested at 24-h intervals was assessed via titration in triplicate in CK cells. Each point represents the mean of three independent experiments, with error bars denoting the SEM. Statistical differences were assessed using a two-way ANOVA with Tukey for multiple comparisons. On all graphs, statistical differences (*P *< 0.05) were identified between M41-K and M41-R at 24 to 96 hpi (not highlighted). Highlighted differences (*) are between the test rIBV and M41-K at 24 to 72 h (B) and 24 to 96 h (C to E) and between the test rIBV and M41-R at 96 hpi (B) and 24 to 96 hpi (F to H).

To determine which of the amino acid differences between M41-K and M41-R are responsible for the *ts* phenotype observed for M41-R and whether these are the same as those involved in pathogenicity, the growth kinetics at 41°C of rIBVs detailed in Table 1 were investigated (Fig. 2). The replication of each rIBV was compared to both M41-K, in which the *in vitro* replication is not affected by the increased temperature, and to M41-R, which exhibits reduced replication at 41°C. Initially, the rIBVs M41-K_Nsp10-L85_ and M41-K_Nsp14-L393-Nsp15-I183-Nsp16-I209_ were investigated to determine whether the single M41-R-derived amino acid in Nsp 10 L85, or the three M41-R-derived amino acids in Nsp 14 L393/Nsp15 I183/Nsp16 I209 were responsible for the *ts* phenotype associated with M41-R. Both rIBVs exhibited reduced titers in comparison to M41-K from 24 to 72 hpi (*P* < 0.05, Fig. 2A and B); although the titers were higher than those observed for M41-R, statistical significance was not reached. This suggests that the presence of either Leu85 in Nsp 10 or the cumulative presence of Leu393, Ile183, or Ile209 in Nsps 14, 15, or 16 is sufficient to result in the *ts* phenotype associated with M41-R, implying that the *ts* phenotype involves both leucine 85 in Nsp 10, along with one or more of M41-R-associated amino acids in Nsps 14, 15, or 16.

The cumulative presence of Leu393, Ile183, and Ile209 in Nsp 14, 15, and 16 was further investigated. The rIBVs M41-K_Nsp14-L393-Nsp16-I209_, M41-K_Nsp14-L393-Nsp15-I183_, and M41-K_Nsp14-L393_, which all contain Pro85 in Nsp 10, all resulted in comparable growth kinetics as M41-R at 41°C (Fig. 2C to E). All of these rIBVs contain the M41-R-associated amino acid Leu393 in Nsp 14; in contrast, both M41-CK and M41-K contain Val393 in Nsp 14, suggesting that the presence of Leu393 in Nsp 14 may be involved in the *in vitro ts* phenotype associated with M41-R. Supporting this suggestion, rIBVs M41-K_Nsp15-I183-Nsp16-I209_, M41-K_Nsp16-I209_, and M41-K_Nsp15-I183_—which all contain Pro85 in Nsp10 and Val 393 in Nsp 14, matching both M41-CK and M41-K—exhibited increased replication at 41°C in comparison to M41-R and comparable growth kinetics to M41-K (Fig. 2F to H).

Overall, although our results indicated that leucine at residue 393 in Nsp 14, rather than a valine, is sufficient to result in a *ts* phenotype. The presence of the amino acid leucine at residue 85, instead of a proline, in Nsp 10 also caused a reduction in replication at 41°C, indicating that the amino acid change in Nsp 10 has a partial *ts* phenotype compared to the effect of leucine at residue 393 in Nsp 14 or when both amino acid changes were present in M41-R. Interestingly, our previous finding showed that these two amino acid changes, either alone or in combination, resulted in *in vivo* attenuation (30), suggesting that these two phenotypes are interlinked and that M41-R, which contains both amino acid changes, would be a more appropriate vaccine candidate than an rIBV which contained only the amino acid change in Nsp 14.

### The replication of rIBV M41-R is increased in comparison to rIBV Beau-R at 41°C.

**FIG 3:**
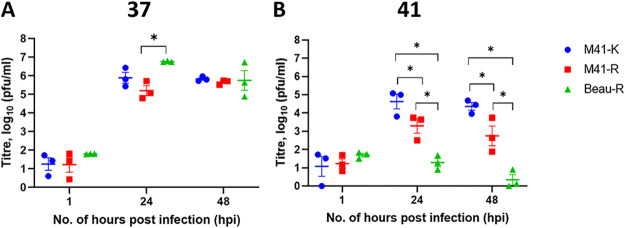
*In vitro* replication of M41-R results in higher quantities of infectious progeny in comparison to Beau-R at 41°C. Primary CK cells were inoculated with 10^4^ PFU (MOI ~ 0.01) of either M41-K, M41-R or Beau-R and incubated at either 37°C (A) or 41°C (B). The quantities of infectious progeny in the supernatant harvested at 1, 24, and 48 hpi were determined via titration in triplicate on CK cells. Each point represents the average (mean) of three independent experiments, with error bars indicating the SEM. Statistical differences were evaluated with a two-way ANOVA with a Tukey test for multiple comparisons and are indicated by an asterisk (*, *P* < 0.05).

The demonstration that the *in vitro* replication of M41-R is *ts* and that this phenotype can be linked to the same amino acid substitutions in Nsps 10 and 14 that resulted in attenuation further highlights the potential for rIBV M41-R as a vaccine candidate. Temperature-sensitive vaccines viruses have been utilized for several viral pathogens, including influenza (29) and mumps (32). The identification that the replication of M41-R is *ts* raises the concern that vaccination with M41-R may not offer more efficacy than vaccination with Beau-R, an rIBV that is also *ts* (28), although it should be noted that rIBV Beau-R does not contain either leucine 85 or leucine 393 in Nsps 10 and 14, respectively. Previous research highlighted that rIBV Beau-R is unable to replicate productively at 41°C (28) and hypothesized that this may be a potential reason why vaccines based on the Beaudette genome does not offer complete protection (17). However, rIBV Beau-R is genetically derived from Beaudette CK, a well-recognized attenuated IBV strain (25), whereas rIBV M41-R is derived from M41-CK, a recognized pathogenic strain (30), indicating that other mutations in the Beaudette genome may be responsible for the lack of efficacy. The data presented in Fig. 1 and 2 show that rIBV M41-R can initiate and sustain limited replication at 41°C, suggesting the possibility of a higher level of replication than is achieved by rIBV Beau-R at this temperature (28). To directly compare the replication of rIBVs M41-R and Beau-R, the titers of infectious progeny produced in primary CK cells at 24 and 48 hpi was investigated (Fig. 3). At 37°C and 48 hpi, the titers were comparable between M41-K, M41-R, and Beau-R. At 41°C, as expected, the titers of both M41-R and Beau-R were reduced in comparison to M41-K (*P* < 0.05). However, the quantities of infectious progeny virus generated from M41-R-infected cells were higher than those for Beau-R (*P* < 0.05) at both 24 and 48 hpi. This suggests that M41-R is able to initiate and sustain replication at 41°C at a higher level (~100-fold) than Beau-R, indicating that M41-R may offer improved *in vivo* replication and potential as a vaccine candidate in comparison to Beau-R.

### A reduction in RNA synthesis at 41°C is observed from 16 hpi.

**FIG 4:**
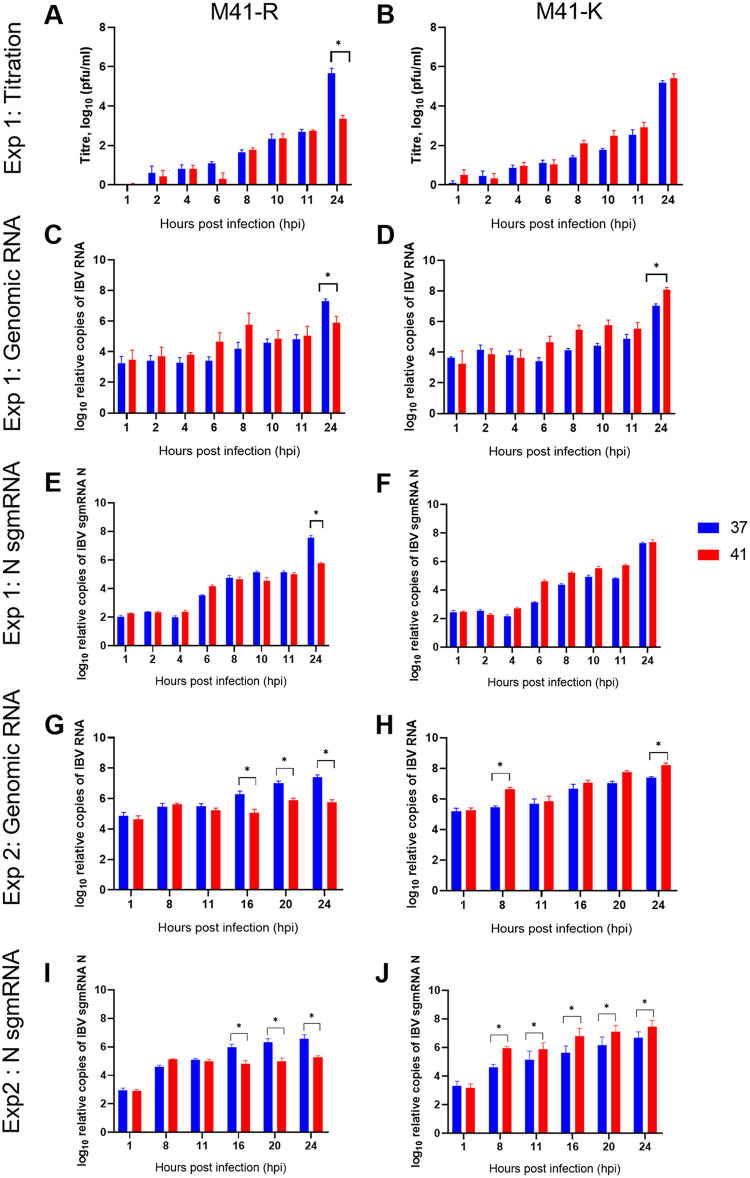
Increased temperature results in reduced RNA synthesis during M41-R infection in CK cells. In experiment 1 (A to F) and experiment 2 (G to J), primary CK cells seeded in six-well plates were inoculated with 10^4^ PFU (MOI ~ 0.01) of either M41-R (A, C, E, G, and I) or M41-K (B, D, F, H, and J) or mock infected with BES medium and incubated at either 37 or 41°C. Cell lysates and cell supernatant were harvested at defined intervals. The quantity of infectious progeny (A and B) in the supernatant was determined via titration in CK cells. Cell lysate was assessed by RT-qPCR for the total quantity of IBV derived genomic RNA (C, D, G, and H) and the quantity of subgenomic RNA (sgRNA) for the nucleocapsid protein (E, F, I, and J), as described previously (74). (A to J) The means of three independent experiments are presented, with error bars representing the SEM. Statistical differences (*, *P* < 0.05) were evaluated using a two-way ANOVA, with a Tukey test for multiple comparisons.

Previous research had demonstrated that the quantity of RNA produced during infection of CK cells at 41°C with Beau-R was reduced from 6 hpi, and this translated to a reduction in infectious progeny virus from 8 hpi (28). To further investigate whether M41-R could offer improved replication *in vivo* in comparison to Beau-R, a growth kinetic assay was carried out focusing on early replication. Both the production of infectious progeny M41-R virus and production of viral RNA were assessed (Fig. 4). No differences in quantities of infectious progeny for M41-R or M41-K were identified at the two temperatures from 1 to 11 h hpi (Fig. 4A and B). As expected, at 24 hpi, M41-R exhibited a decrease in the quantity of infectious progeny at 41°C. The amounts of IBV-derived genomic RNA and subgenomic mRNA for N, for both viruses, were also comparable between 1 and 11 hpi, suggesting that temperature did not affect RNA synthesis during the early stages of replication (Fig. 4C to F). During M41-R infection, a reduction in the quantity of IBV-derived RNA, both genomic and subgenomic, was observed at 24 hpi at 41°C in comparison to 37°C. This suggests that in subsequent rounds of replication, with the IBV replication cycle reported to be between 6 and 8 h (33), M41-R RNA synthesis is affected by temperature. To further investigate this, a second experiment was carried out investigating additional time points (Fig. 4G to J). A reduction in RNA synthesis during M41-R infection at 41°C, both genomic and subgenomic, was observed from 16 hpi (Fig. 4G and I). Interestingly, the quantity of N subgenomic mRNA (sgmRNA) during M41-K infection was greater in comparison to 37°C from 8 hpi; (Fig. 4J) this, however, was only observed for genomic RNA at 8 and 24 hpi (Fig. 4H).

### M41-R infection of CK cells at 41°C results in a higher upregulation of IFN-β relative to lower viral load.

**FIG 5:**
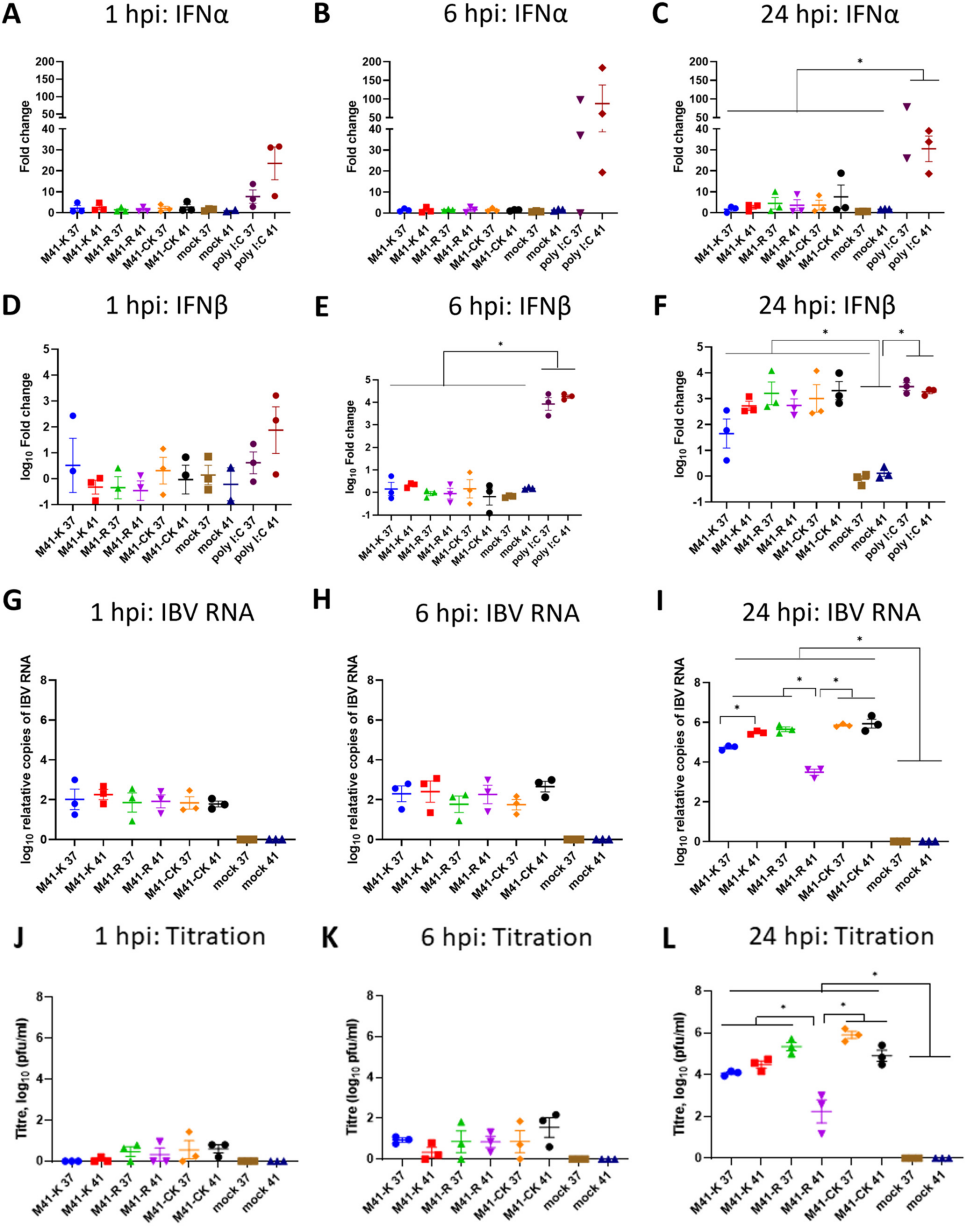
M41-R infection of CK cells at 41°C results in an upregulation of IFN-β relative to lower quantities of viral RNA and infectious progeny. Primary CK cells seeded in six-well plates were inoculated with 10^4^ PFU of either M41-K, M41-R, or M41-CK or mock infected with BES medium. Poly(I:C) was used as an inducer of host response; cells received 1 μg. Cells were incubated at either 37 or 41°C with cell lysate harvested at 1, 6, and 24 hpi. RNA was extracted from the cell lysate and analyzed by qRT-PCR for IFN-α (A to C) and IFN-β (D to F). All data were normalized to RPL13, and fold changes were calculated compared to mock-infected cells. (G to I) The quantity of IBV-derived RNA was investigated using primers and probes targeting the 5′ UTR (74). (J to L) The quantity of infectious progeny was determined via titration in triplicate in CK cells. Statistical differences were investigated using a one-way ANOVA with a Tukey test for multiple comparisons and are indicated by an asterisk (*, *P* < 0.05).

**FIG 6:**
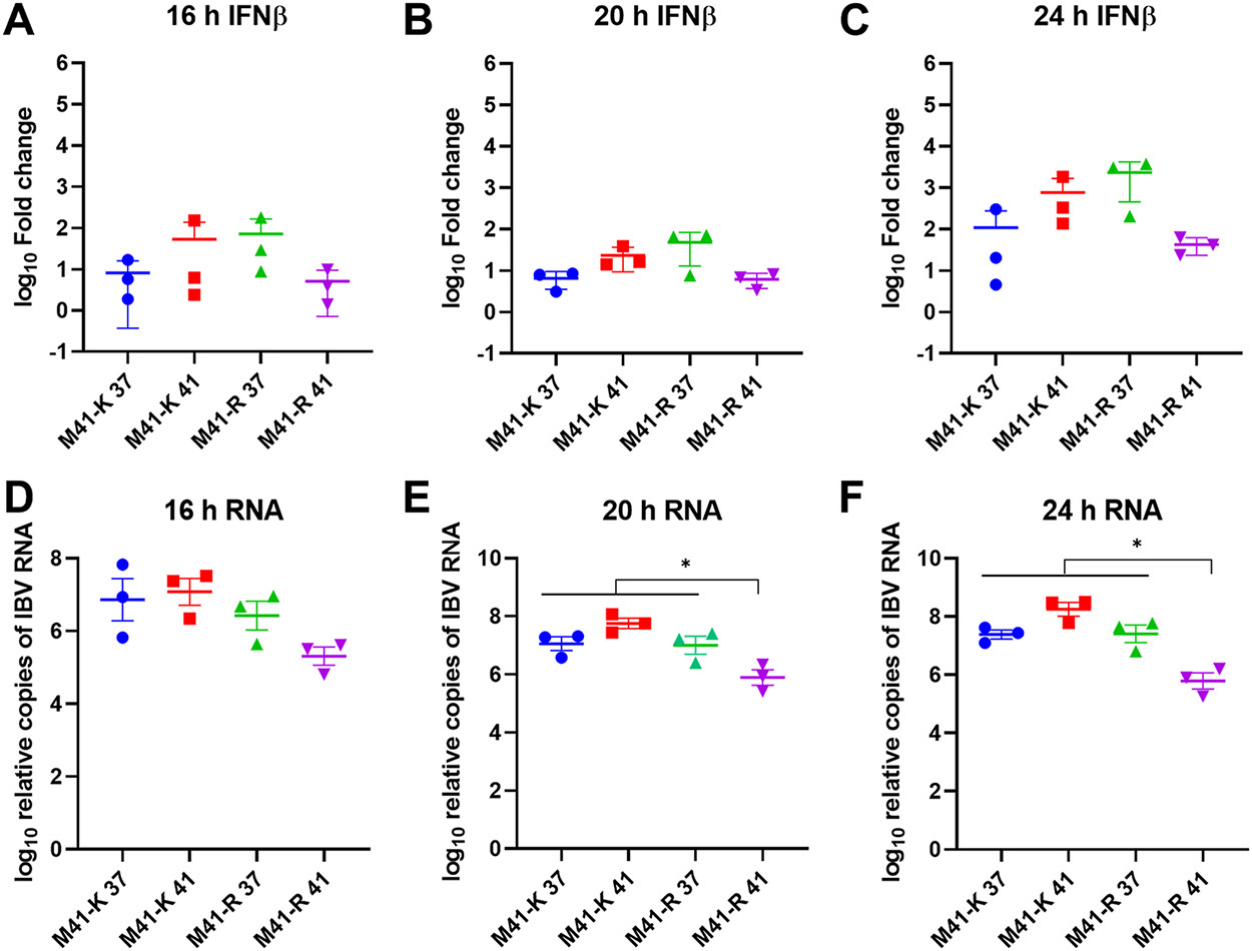
M41-R infection of CK cells at 41°C results in an upregulation of IFN-β relative to lower quantities of viral RNA. Primary CK cells seeded in six-well plates were inoculated with 10^4^ PFU of either M41-K, M41-R, or M41-CK or mock infected with BES medium. Cells were incubated at either 37 or 41°C with cell lysate harvested at 16, 20, and 24 hpi. (A to C) RNA was extracted from the cell lysate and analyzed by qRT-PCR for IFN-β. Values were normalized to RPL13, and fold changes were calculated compared to mock-infected cells. (D to F) The quantity of IBV-derived RNA was investigated using primers and probes targeting the 5′ UTR (74). Error bars represent the SEM of three biological repeats, and statistical differences—indicated by an asterisk (*, *P* < 0.05)—were investigated using a one-way ANOVA with a Tukey test for multiple comparisons.

Recent research has indicated that *in silico*-derived mutations to affect the N7-methyltransferase activity associated with the coronavirus Nsp 14 resulted in the upregulation of type I interferon (IFN-I) and the loss of viral evasion against the IFN-I-mediated immune response as a result of reduced guanine N7-methylation of the 5′ cap on the viral RNAs (34). To determine whether the amino acid responsible for the *ts* phenotype of M41-R in Nsp 14 had any effect on the IFN-I-mediated immune response, which has been shown previously to be modulated during IBV infection (35, 36), the upregulation of IFN-α and IFN-β, as determined by quantitative real-time PCR (qRT-PCR), was assessed during infection of primary CK cells with either M41-K, M41-R, or the parental wild-type virus M41-CK at both 37 and 41°C (Fig. 5). At 1, 6, and 24 hpi, IFN-α did not appear to be upregulated by either M41-R, M41-K, or M41-CK infection at either temperature (Fig. 5A to C). IFN-β was upregulated at 24 hpi, though there was no significant difference between the viruses investigated or the temperature (Fig. 5D to F). Although the quantity of IFN-β appeared greater during M41-K infection at 37°C than during M41-R infection, statistical significance was not reached. Previous research has associated higher levels of IFN-β expression with lower infectious progeny (36). Both the quantity of M41-K and M41-CK RNA and infectious progeny were comparable between 37 and 41°C (Fig. 5G to L); however, the quantity of IBV-derived RNA in the M41-R-infected cells (Fig. 5G to I) and the quantity of infectious progeny (Fig. 5J to L) were lower at 41 than 37°C. This was expected from the results presented in Fig. 4 and was notably significantly lower in comparison to M41-K at 41°C. A second experiment was carried out investigating IFN-β expression at additional time points (Fig. 6). At 16, 20, and 24 hpi, the levels of IFN-β expression were comparable (Fig. 6A to C); however, at both 20 and 24 hpi, the quantity of M41-R RNA (Fig. 5E and F) at 41°C was lower, in line with the findings displayed in Fig. 5. The results displayed in Fig. 5 and 6 therefore suggest that the amino acid changes present in M41-R in comparison to both M41-K and M41-CK impacted the IFN-β response to viral infection *in vitro* at 41°C.

### Tracheal ciliary activity is retained after vaccination with M41-R.

**FIG 7:**
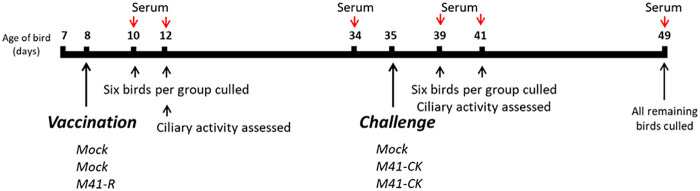
Schematic detailing protocol for *in vivo* vaccine challenge experiment. Groups of 8-day-old SPF RIR chickens were vaccinated by the intraocular/intranasal route with either 10^4^ PFU of rIBV M41-R or mock vaccinated with PBS. At 27 days p.v., the birds were challenged with either 10^4^ PFU of M41-CK or mock challenged with PBS. Clinical signs, including tracheal rales and snicking, were assessed 2 to 7 days both p.v. and p.c. At defined intervals, randomly chosen birds were culled from each group, and a variety of tissues were harvested. Serum was collected both postvaccination (prechallenge) and postchallenge. Tracheal ciliary activity was assessed at day 4 p.v. and at days 4 and 6 p.c. All remaining birds were culled 14 days p.c. for tissue and serum collection.

**FIG 8:**
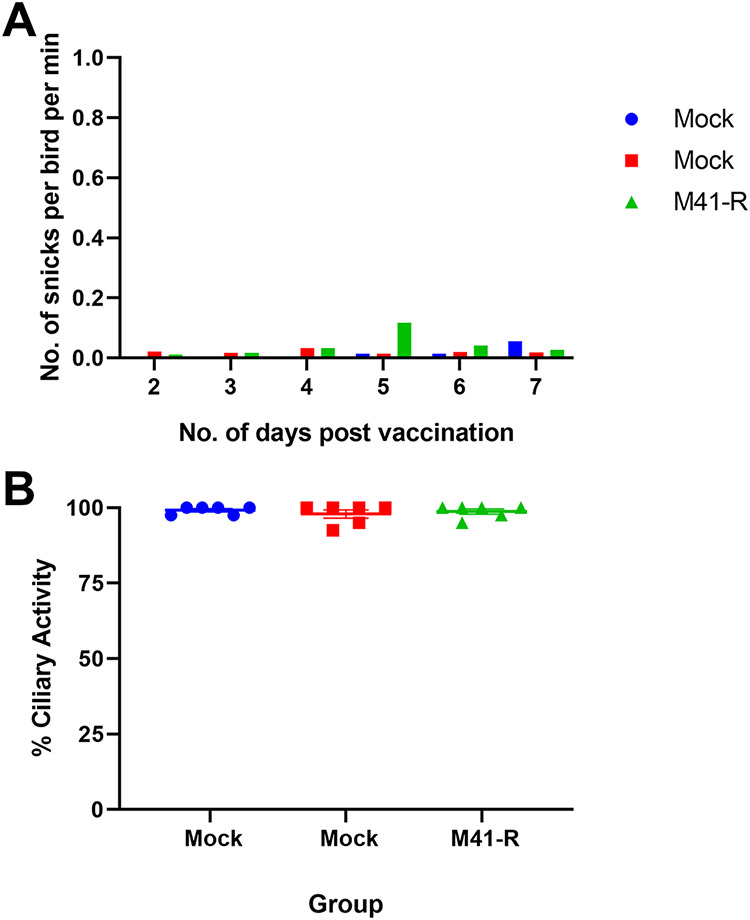
Vaccination with M41-R does not result in IBV-induced clinical signs or a reduction in tracheal ciliary activities. At 8 days of age, SPF chickens were vaccinated with 10^4^ PFU of M41-R or mock vaccinated with PBS. (A) The number of snicks in each group was assessed from days 2 to 7 days p.v. Over a 2-min period, the numbers of snicks per group were independently counted by two or three persons with the average (mean) of these scores calculated as snicks per bird per minute for each group. (B) At 4 days p.v., the tracheas were harvested from six randomly sampled birds; each trachea was sectioned in 10 × 1 mm rings. The ciliary activity of each ring was assessed by light microscopy, and the percent activity was calculated. Plotted points represent individual birds, and the mean activity of the 10 rings was assessed. Error bars represent the SEM. Statistical differences were assessed using a one-way ANOVA with a Tukey test for multiple comparisons; no differences were identified.

To establish whether vaccination with M41-R could induce a protective immune response against homologous challenge with pathogenic M41-CK, three groups of 8-day-old specific-pathogen-free (SPF) Rhode Island Red (RIR) chicks were inoculated with either M41-R or phosphate-buffered saline (PBS) for mock vaccination (Fig. 7). The levels of snicking observed for the M41-R inoculated chicks postvaccination (p.v.) were comparable to the mock-vaccinated birds (Fig. 8A), and no rales were observed in any of the groups, demonstrating that M41-R did not result in clinical disease, as expected from previous research (30). The ciliary activities of the tracheal epithelial cells were assessed in six randomly chosen birds from each group at day 4 p.v. (Fig. 8B). From each trachea harvested, 10 × 1 mm rings were sectioned (three from the top of the trachea, four from the middle, and three from the bottom), and the ciliary activities assessed by light microscopy (37, 38). The average activities were comparable between both mock-vaccinated groups and those vaccinated with M41-R: 99.2, 97.9, and 98.8%, respectively. No individual tracheal ring score under 75% ciliary activity in birds infected with M41-R, comparable to both mock-infected groups. Vaccination with M41-R therefore had no effect on tracheal ciliary activity. This result, alongside the lack of clinical signs, verified that M41-R displayed an attenuated *in vivo* phenotype, as previously observed (30).

### IBV RNA was detected in eyelid tissue from M41-R-vaccinated chickens.

Tracheas, eyelids, and beaks were harvested from six randomly chosen birds from each group on days 2 and 4 p.v., and the tissues assessed for the presence of IBV-derived RNA by RT-PCR specifically targeting the 3′-untranslated region (3′ UTR). On days 2 and 4 p.v., no IBV-derived RNA was identified in beak or tracheal tissue, and the lack of virus isolation from tracheal tissue supported this observation (Table 2). Interestingly, at day 4 p.v., the eyelids extracted from four of six birds in the M41-R-vaccinated group were positive for IBV RNA by RT-PCR, suggesting the presence of rIBV M41-R in this tissue. Further analysis was by virus isolation using embryonated hens’ eggs demonstrated that infectious M41-R was present in the eyelid tissue.

**TABLE 2:** Presence of IBV derived RNA and infectious virus postvaccination^*a*^

No. of days p.v.	Inoculum	RT-PCR			Virus isolation	
		Eyelid	Beak	Trachea	Eyelid	Trachea
2	Mock	0/6	0/6	0/6	0/6	0/6
	Mock	0/6	0/6	0/6	0/4*	0/6
	M41-R	0/6	0/6	0/6	0/2*	0/6
4	Mock	0/6	0/6	0/6	0/6	0/6
	Mock	0/6	0/6	0/6	0/6	0/6
	M41-R	4/6	0/6	0/6	5/6	0/6

a Virus presence was determined from randomly selected birds culled 2 and 4 days p.v. The results are displayed as the number of positive birds/the total number of birds sampled. RT-PCR was performed on homogenized tissue, using primers targeting the IBV 3′ UTR. Virus isolation was performed in embryonated hens’ eggs, with the resulting allantoic fluid also assessed by RT-PCR targeting the 3′ UTR. *, Some samples were damaged during collection and could not be analyzed.

### M41-R induced a protective immune response against homologous M41-CK challenge.

**FIG 9:**
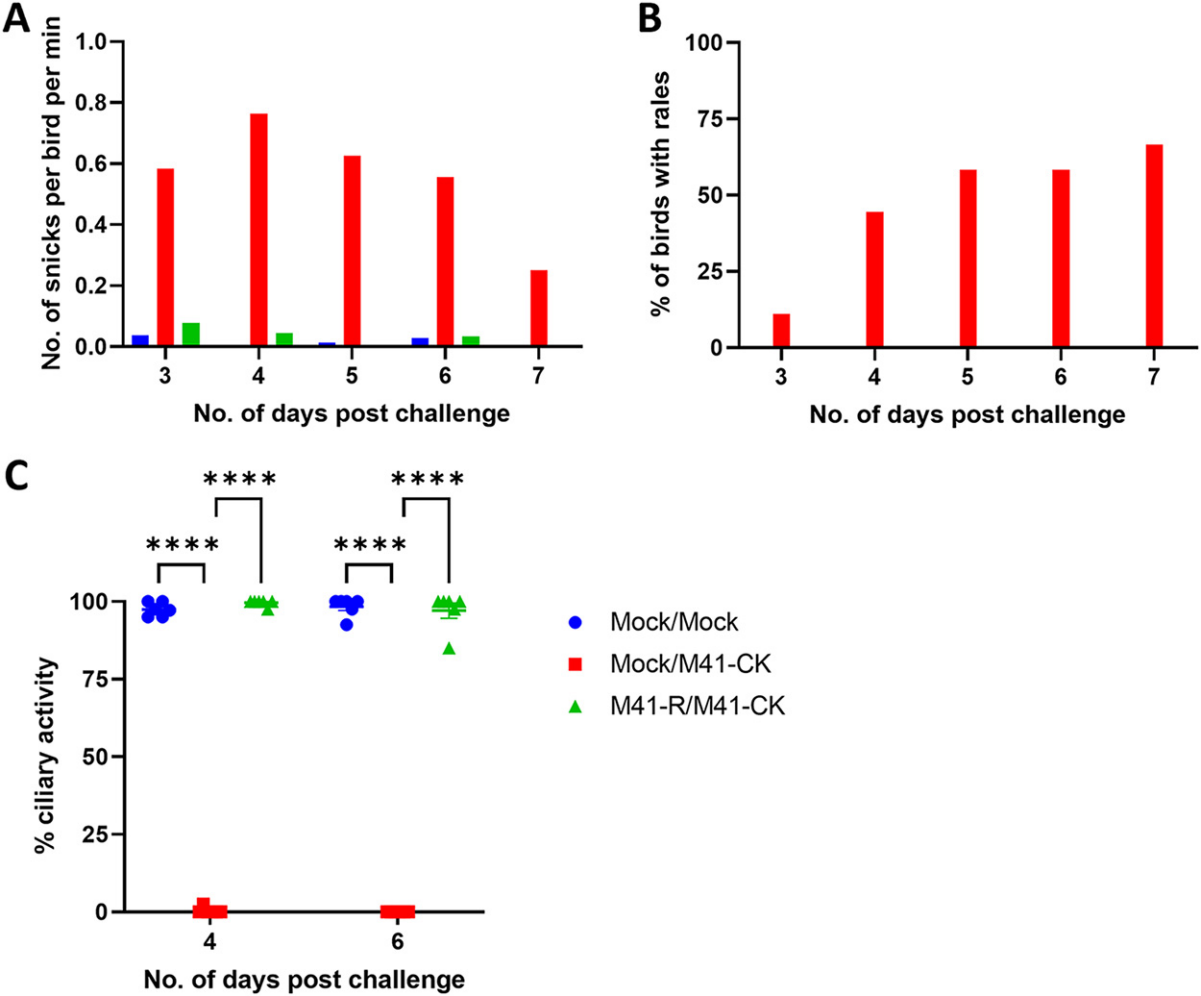
Vaccination with M41-R protects chickens against IBV-induced clinical signs and tracheal damage after challenge with M41-CK. At 27 days p.v., chickens were challenged with either 10^4^ PFU of M41-CK or mock challenged with PBS. (A) The numbers of snicks in each group were assessed from 2 to 7 days postchallenge. Over a 2-min period, the numbers of snicks per group were independently counted by two or three persons with the average (mean) of these scores calculated as snicks per bird per minute for each group. (B) Chickens were individually assessed for tracheal rales from days 2 to 7 p.c. The percentage of birds exhibiting rales in each group was calculated. (C) On days 4 and 6 p.c., the tracheas were harvested from six randomly sampled birds and sectioned into 10 × 1 mm rings. The ciliary activity of each ring was assessed by light microscopy, and the percent activity was calculated. Plotted points represent individual birds, and the mean activity of the 10 rings was assessed, with error bars representing the SEM. Statistical differences were assessed using a two-way ANOVA with a Tukey test for multiple comparisons and are highlighted by **** (*P* < 0.0001).

At 27 days p.v. with M41-R, the remaining chickens in each group were either challenged with M41-CK or treated with PBS as a mock challenge (Fig. 7 and 9). The levels of snicking observed postchallenge (p.c.) were comparable between the M41-R-vaccinated/M41-CK-challenged birds and the mock-vaccinated/mock-challenged birds (Fig. 9A), remaining below 0.04 snicks per bird per min. In contrast, the mock-vaccinated/M41-CK-challenged birds exhibited snicking at a rate greater than 0.5 snicks per bird per min from days 3 to 6 p.c., peaking at 0.76 on day 4 p.c. Rales were not observed in either the mock-vaccinated/mock-challenged or the M41-R-vaccinated/M41-CK-challenged group (Fig. 9B). In contrast, rales were observed in the mock-vaccinated/M41-CK-challenged group, peaking at 66.7% on day 7 p.c. The assessment of snicking and rales therefore demonstrated that M41-R vaccination resulted in protection against IBV M41-CK-induced clinical signs.

Tracheas and eyelid tissues were harvested from six randomly chosen birds per group on days 4 and 6 p.c. RT-PCR analysis could not detect IBV-derived RNA in any of the tissues harvested from the mock-vaccinated/mock-challenged group or from the M41-R-vaccinated/M41-CK-challenged group, suggesting that M41-R vaccination induced protection against M41-CK challenge. On day 4 p.c., all tracheal and eyelid tissues from birds in the mock vaccination/M41-CK challenge group were positive for the presence of IBV-derived RNA (Table 3). Virus isolation from the tracheas and eyelids confirmed that no infectious M41-CK virus was present in M41-CK-infected birds previously vaccinated with M41-R, suggesting that vaccination with M41-R induced protection against M41-CK infection.

**TABLE 3:** Presence of IBV derived RNA and infectious virus postchallenge^*a*^

No. of days p.c.	Vaccination/challenge	RT-PCR		Virus isolation	
		Eyelid	Trachea	Eyelid	Trachea
4	Mock vaccinated/Mock challenged	0/6	0/6	0/6	0/6
	Mock vaccinated/M41-CK challenged	6/6	6/6	6/6	5/6
	M41-R vaccinated/M41-CK challenged	0/6	0/6	0/6	0/6
6	Mock vaccinated/Mock challenged	0/6	0/6	0/6	0/6
	Mock vaccinated/M41-CK challenged	6/6	0/6	2/6	0/6
	M41-R vaccinated/M41-CK challenged	0/6	0/6	0/6	0/6

a Virus presence was determined from randomly selected birds culled 4 and 6 days p.c. The results are displayed as the number of positive birds/the total number of birds sampled. RT-PCR was performed on homogenized tissue, using primers targeting the IBV 3′ UTR. Virus isolation was performed in embryonated hens’ eggs, with the resulting allantoic fluid also assessed by RT-PCR targeting the 3′ UTR.

In line with the requirements of the *European Pharmacopoeia* (2020) (39), tracheal ciliary activities were assessed on days 4 and 6 p.c., with the values observed comparable between the mock vaccinated/mock-challenged and M41-R-vaccinated/M41-CK-challenged groups. The average ciliary activities on days 4 and 6 p.c. were 97.5 and 98.3% and 99.7 and 97.2%, respectively (Fig. 9C). In contrast, the average ciliary activities observed from the mock-vaccinated/M41-CK-challenged group were 0.4% on day 4 p.c. and 0% on day 6 p.c. Vaccination with M41-R therefore induced protection against the loss of tracheal ciliary activity. The *European Pharmacopeia* (39) states that for a bird to be classified as fully protected at least 50% ciliary activity must be retained postchallenge in 9 of 10 tracheal rings sampled; all sampled M41-R-vaccinated/M41-CK-challenged birds on both days 4 and 6 p.c. met this requirement. This, alongside the lack of IBV-induced clinical signs, demonstrates that vaccination with M41-R can induce a protective response against a homologous M41-CK challenge. In addition, the lack of detection of either challenge virus-derived RNA or infectious challenge virus suggests that vaccination with M41-R elicited complete protection, at least as determined by the diagnostic tests of this study, against M41-CK.

### Vaccination with M41-R induces a robust antibody response.

**FIG 10:**
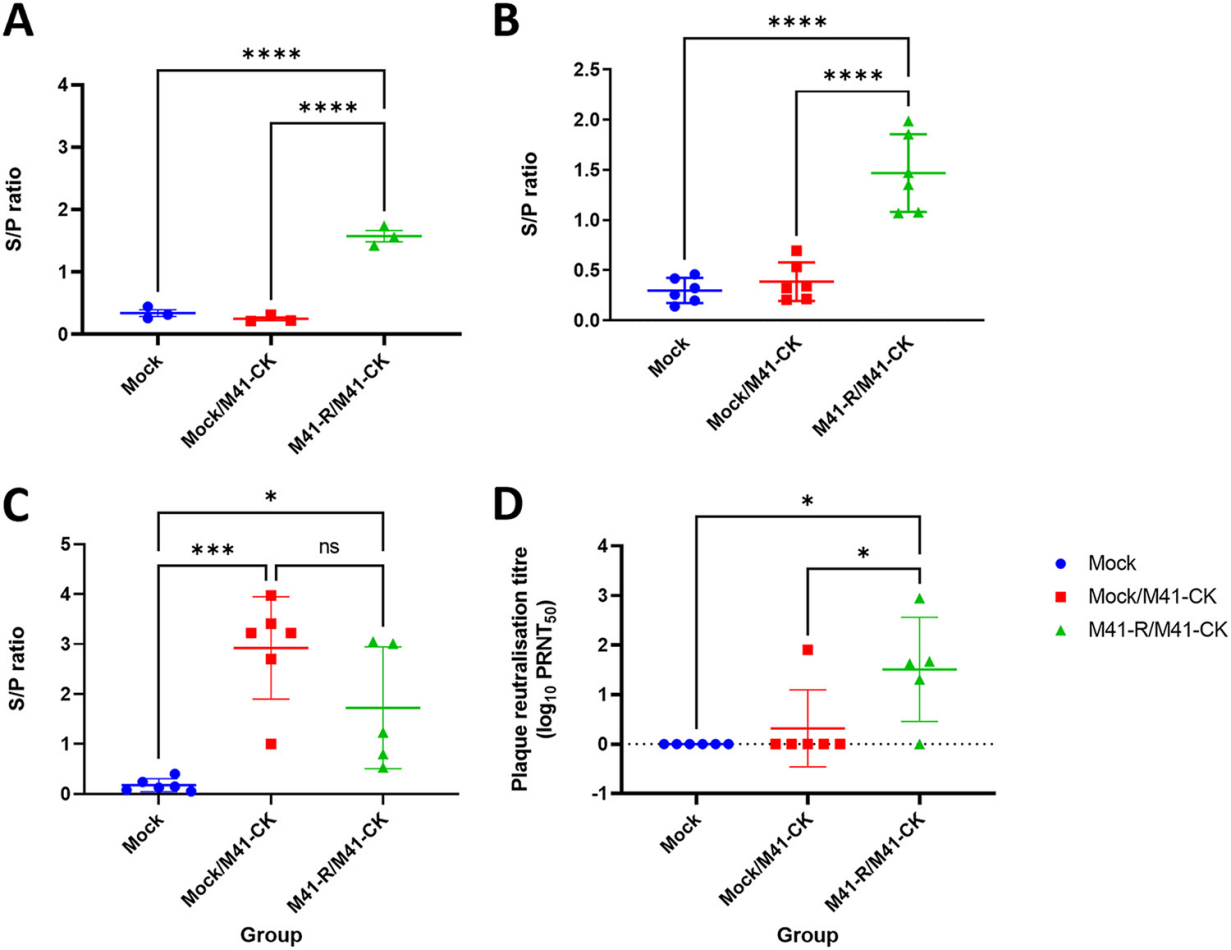
Vaccination with M41-R induces a robust antibody response. Serum was harvested from chickens on day 14 p.v. (A) and days 4 (B) and 14 p.c. (C) and diluted 1/80 for ELISA using the commercial BioChek IBV ELISA kit. Samples were run in triplicate, and the average S/P ratio was calculated. S/P ratios are displayed with error bars representing the standard deviations (SD). Statistical differences were assessed by one-way ANOVA, followed by a Tukey test for multiple comparisons. (D) To assess the levels of neutralizing antibody, serum from day 14 p.c. was serially diluted and incubated with 1,000 PFU of M41-CK, followed by a plaque assay on CK cells to determine the PRNT_50_. The PRNT_50_ values were calculated from at least two biological repeats for each group using the Reed-Muench method. Average PRNT_50_ values for each bird are displayed, with error bars representing the SD. Statistical differences were assessed by one-way ANOVA, followed by a Tukey test for multiple comparisons and are highlighted by * (*P* < 0.05), *** (*P* < 0.0005) and **** (*P* < 0.0001).

To assess whether vaccination with M41-R had induced a humoral antibody response, serum was assessed for the presence of IBV-specific antibodies both p.v. and p.c. by enzyme-linked immunosorbent assay (ELISA) (Fig. 10). The levels of IBV-specific antibody in prechallenge samples (day 14 p.v.) were significantly higher among M41-R-vaccinated birds than in either of the mock-vaccinated groups (*P* < 0.001), demonstrating that vaccination with the rIBV M41-R induced an IBV-specific humoral response (Fig. 10A). At 4 days p.c. with M41-CK, IBV-specific antibody levels were significantly higher among M41-R-vaccinated birds compared to mock-vaccinated birds, with a mean S/P ratio of 1.5 (Fig. 10B). There is variation in the values for each bird, suggesting variation in the response to vaccination. At day 14 p.c., increased levels of IBV-specific antibody are observed in both mock/M41-CK and M41-R/M41-CK groups compared to the mock group, with variation between individual birds observed for both challenged groups (Fig. 10C). There was no significant difference between the mock/M41-CK and M41-R/M41-CK groups, although the average S/P ratio was higher in the mock/M41-CK group, showing that M41-R induced a comparable antibody response to infection with IBV after 14 days.

Serum neutralizing antibody levels at day 14 p.c. were assessed by determining the plaque reduction neutralization titer (PRNT_50_) for each bird. Serial dilutions of serum samples were incubated with 1 × 10^3^ PFU of M41-CK and titrated on CK cells to assess the reduction in viral titer after incubation with sera. The PRNT_50_ values for each bird in each group are displayed in Fig. 10D. As expected, no neutralizing antibody against M41-CK was detected in the mock group. Neutralizing antibody was only detected in one bird in the mock/M41-CK-challenged group. In the M41-R/M41-CK-challenged group, neutralizing antibody was detected in sera harvested from four of the five birds. Interestingly, serum harvested from one bird displayed no neutralization activity, despite the bird exhibiting complete protection from M41-CK-induced clinical signs and ciliostasis (Fig. 9).

Taken together, the results from the assessment of serum antibody levels indicate that M41-R induces a robust antibody response following vaccination, including the induction of neutralizing antibodies against the challenge virus, further demonstrating its potential as a promising IBV vaccine candidate.

## DISCUSSION

Temperature sensitivity, particularly in viruses causing respiratory disease, has been used to study gene function and mechanisms of attenuation for several viruses, including mumps virus (32), human parainfluenza virus (40, 41), Respiratory syncytial virus (42), and influenza virus (30). The latter is particularly well researched with temperature-sensitive mutants used for not only vaccination but also the identification of genes involved in viral replication and pathogenesis. Temperature sensitivity has been used in studies investigating the coronavirus replication cycle in which *ts* mutants were used to elucidate specific roles for the Nsps (43). Temperature-sensitive mutants have also been used to elucidate protein-protein interactions, as well as RNA-protein interactions (44–47). Temperature has also been demonstrated to play a role in coronavirus entry, with the receptor-binding domain of SARS-CoV-2 showing increased affinity with the cellular receptor, angiotensin-converting enzyme 2 (ACE2), at lower temperatures (48, 49).

For all animals, including avian species, continuous movement of air in the respiratory tract cools this area in comparison to the core of the body. For birds, this means the upper respiratory tract, including the nasal tissue, beak, and throat, is cooler (37 to 38°C) than the lower respiratory tract, including the trachea and lungs. Previous research into IBV has identified that the *in vitro* replication of the attenuated rIBV Beau-R is sensitive to temperature and is highly restricted at 41°C, the core body temperature of a chicken (28). Beau-R has been extensively researched as a live-attenuated vaccine vector with the replacement of the spike gene with either M41-CK or 4/91(UK) inducing a protective response against homologous challenge (17, 26, 27). Protection at ~65% as defined by ciliary activity is not sufficient under the standards set by the *European Pharmacopeia* (39), and it was hypothesized that this may be a consequence of restricted replication *in vivo*, which is likely the result of temperature sensitivity (17, 28).

In this study, we show that *in vitro* replication of the attenuated rIBV M41-R is reduced at 41°C in comparison to 37°C (Fig. 1); however, it is not as restricted as rIBV Beau-R (Fig. 3). This may suggest that the *ts* phenotype associated with M41-R is not due to same mechanism as Beau-R. The replication of the pathogenic rIBV M41-K is not affected by temperature. Four amino acid residues—Pro85Leu, Val393Leu, Leu183Ile, and Val209Ile in Nsps 10, 14, 15, and 16, respectively—distinguish M41-K from M41-R (30). We identified that the change the Val393Leu in Nsp 14 gave a *ts* phenotype similar to that observed for M41-R and that the change Pro85Leu in Nsp 10 also caused some reduction in replication at 41°C (Fig. 2). This observation indicates that the *ts* phenotype observed by M41-R is potentially caused as a result of the amino acid change in Nsp 14 and that this effect essentially masks the *ts* phenotype observed by the change in Nsp 10. Our previous research demonstrated that the same changes resulted in attenuation of M41-R (30). We conclude, with regard to these specific residues, that temperature sensitivity and pathogenicity are linked. While our previous research into the *ts* replication phenotype of different IBV strains with various *in vivo* pathogenic phenotypes could not definitely link temperature sensitivity and attenuation, it was observed that all the pathogenic isolates investigated were able to replicate at 41°C (28). In addition, there is precedent for the linkage of temperature sensitivity and attenuation, with mutations in Nsp 3 rendering the *in vitro* replication of murine hepatitis virus (MHV) temperature sensitive, as well as resulting in an attenuated *in vivo* phenotype in mice (47).

The amino residues responsible for the *ts* phenotype associated with M41-R in this study are located in Nsps 10 and 14. Nsp 14 is a bifunctional protein consisting of both a 3′–5′ exoribonuclease (EXON) and an *S*-adenosylmethionine (SAM)-dependent (guanine-N7) methyltransferase (N7-MTase) (50, 51). Nsp 10 is a small 15-kDa protein (52, 53) that acts as a cofactor for the EXON activity of Nsp 14 (54, 55), as well as Nsp 16, a 2′-*O*-methyltransferase (56–58). Temperature-sensitive isolates of MHV have been reported with the individual mutations Gln65Glu in Nsp 10 and Cys376Tyr and Cys408Arg in Nsp 14 (59). The expression of Nsp 14 in yeast containing the equivalent mutations in SARS-CoV also led to a *ts* phenotype with the hypothesis that the mutations had destabilized the N7-MTase domain at the higher temperature (60). However, analysis of the change in the predicted Gibbs free energy (*G*) of Nsp 10 containing the Pro85Leu change and Nsp 14 containing the Val393Leu change in M41-R using I-Mutant 2.0 (https://folding.biofold.org/i-mutant/i-mutant2.0.html) (61) does not suggest a decrease in protein stability at 41°C in comparison to 37°C (Table 4).

**TABLE 4:** Change in entropy in Nsps 10 and 14 between 37 and 41°C^*a*^

Nsp	Residue	M41-K	M41-R	pH	Temp (°C)	ΔΔ*G*		ΔΔ*G* difference	
						kcal/mol	kJ/mol	kcal/mol	kJ/mol
10	85	Pro	Leu	6.0	37	–1.45	–6.0668	–0.05	–0.21
					41	–1.40	–5.8576		
				6.5	37	–1.41	–5.8994	–0.04	–0.17
					41	–1.37	–5.7321		
14	393	Val	Leu	6.0	37	–1.94	–8.1170	–0.02	–0.08
					41	–1.92	–8.0333		
				6.5	37	–1.96	–8.2006	–0.02	–0.08
					41	–1.94	–8.1170		

a Predicted changes in protein stability using Gibbs free energy (*G*) at 41°C in comparison to 37°C at both pH 6.0 and 6.5. Values were obtained using I-mutant 2.0 (61; https://folding.biofold.org/i-mutant/i-mutant2.0.html).

How the changes in both Nsp 10 and 14 have resulted in both a *ts* phenotype, as well as an attenuated phenotype, remains an important avenue of future research. Recent work on MHV identified two mutations, D330A and Y141A, in the N7-MTase domain in Nsp 14 of MHV that resulted in attenuation (62). Similarly, the equivalent mutations in SARS-CoV-2 were found to be attenuating in transgenic mice (34). There have been publications that have investigated the critical residues required for N7-MTase function (60, 63–65); the residue equivalent to 393 in IBV is not reported to be one of them. The Val393 residue is located in the N7-MTase domain between motifs V and VI of the conserved residues identified in the catalytic pocket (66). Both motifs V and VI are reported to be RNA-binding motifs. Interestingly, it was previously reported that the function of the EXON and N7-MTase domains are functionally independent (64); however, recent research by Ogando et al. suggests this may not be the case (66). Mutations in the hinge region of the N7-MTase domain of MERS-CoV were found to affect EXON activity, with the authors hypothesizing that this was either the result of the mutations affecting overall folding of Nsp 14, or the mutations had constrained the hinge subdomain negatively affecting EXON activity. The Val393 residue is located 12 amino acid residues upstream of one of the hinge subdomains previously reported (65). It should be noted that the effect of temperature on the replication of the recombinant viruses was not assessed in any of the previously reported studies (34, 60, 62–64, 66).

In our study, RNA synthesis during early replication of M41-R does not appear to have been impacted by temperature; however, it is reduced from 16 hpi (Fig. 4). This may therefore suggest that the effect of changes Pro85Leu (Nsp 10) and Val393Leu (Nsp 14) may not directly impact RNA synthesis and may instead exert the effect either downstream of RNA synthesis or may be host dependent, which consequently impacts RNA synthesis. Temperature has been shown to affect host immune responses to viral infection (67), including SARS-CoV-2 in which temperature-dependent host interferon and proinflammatory responses have been demonstrated (68). The N7-MTase activity of Nsp 14 is involved in the capping of viral RNA (64, 69), which is important for both viral protein production and evasion of the host responses (70). Pan et al. demonstrated that the Tyr414Ala mutation in Nsp14 of MHV resulted in the upregulation of a type I IFN response (34). In this study, however, no differences in either the upregulation of IFN-α or IFN-β in M41-R-infected CK cells at either 37 or 41°C were identified (Fig. 5 and 6); however, it must be noted that at 20 and 24 hpi, the quantities of viral RNA were not comparable. Virus-host responses are dependent on cell type, so it is possible that *in vivo* the IFN response is impacted by amino acid changes present in rIBV M41-R.

Regardless of how the Pro85Leu and Val393Leu changes in M41-R Nsps 10 and 14, respectively, have imparted both a *ts* and an *in vivo* attenuated phenotype, our study showed that a single dose of rIBV M41-R protected chickens against homologous M41-CK challenge in terms of both clinical disease and loss of ciliary activity (Fig. 9). Analysis of the antibody responses both postvaccination and postchallenge demonstrated that vaccination with M41-R induced a robust antibody response, including the development of virus neutralizing antibodies (Fig. 10). Although this matches the observed results of the MHV Nsp 14 mutants (62), the presence of IBV humoral antibody is generally considered unsuitable for the evaluation of vaccine efficacy (71). This is evidenced by the variation in neutralizing antibody responses between birds vaccinated with M41-R, where complete protection was achieved for all birds, despite a lack of neutralizing antibody in one bird and half the amount of antibody in others compared to the bird with the highest PRNT_50_. Assessment of ciliary activity is considered one of the gold standards for assessing IBV vaccine efficacy; the *European Pharmacopoeia* (39) classifies a chicken as protected if 50% ciliary activity (or greater) is retained postchallenge in 9 of 10 tracheal rings. All of the M41-R-vaccinated and M41-CK-challenged birds in this study met this requirement on both days 4 and 6 p.c. (Fig. 9C). In addition, tracheal ciliary activities were retained p.v. (Fig. 8B), demonstrating that M41-R vaccination did not result in any tracheal damage. This is an advantage over the currently used Mass vaccine, H120 (8). Alongside retention in tracheal ciliary activity, the absence of challenge virus is also a beneficial consideration; no infectious virus could be detected postchallenge (Table 3), suggesting that unlike the MHV studies (34, 62), the protective immunity induced by M41-R was sterilizing with respect to detection of M41-CK challenge virus infection. It must, however, be noted that in our study, the first sampling point was day 4 p.c., and it is therefore possible that challenge virus may have been detected earlier but the immunity induced resulted in rapid viral clearance.

As indicated in our previous work (30), the proline at residue 85 in Nsp 10 and the valine at residue 393 in Nsp 14 are conserved among not only IBV strains but also in other members of the coronavirus family. This study highlights that the amino acid changes Pro85Leu and Val393Leu in Nsps 10 and 14 of the M41 strain of IBV impart not only an attenuated *in vivo* phenotype but also a temperature-sensitive replication phenotype. Both of these amino acid changes were identified in a natural isolate and also in an IBV population (30), rather than been generated by *in silico* methods to identify potential amino acids that may affect active sites in a Nsp domain. This may be one reason why changes in two Nsps are required and why this has the potential to be more stable than the introduction of an *in silico*-designed mutation that may not exist in a natural isolate. The double change in both Nsp 10 and 14 may therefore offer an avenue for the development of live-attenuated vaccines against not only other IBV strains but also other coronaviruses.

## MATERIALS AND METHODS

### Cells and viruses.

Primary chicken kidney (CK) cells were prepared from kidneys extracted from 2- to 3-week-old SPF RIR chickens hatched and reared at The Pirbright Institute according to a previously published protocol (72). Cells seeded in tissue culture plates were incubated at 37°C and 5% CO_2_ for 2 days prior to use.

The rIBVs Beau-R (25), M41-K, M41-R, M41R-nsp10rep, M41-R-nsp14.15.16rep, M41R-nsp10.14rep, M41R-nsp10.15rep, M41R-nsp10.16rep, M41R-nsp10.14.15rep, M41R-nsp10.15.16rep, and M41R-nsp10.14.16rep have been described previously (30). The pathogenic laboratory IBV strain M41-CK (GenBank accession number MK728875.1) has also been described previously (10). All viruses were propagated in embryonated hens’ eggs provided by Valo Biomedia (Germany). Allanotic fluid was clarified by low-speed centrifugation, and the quantity of infectious virus was determined via titration in CK cells. All nucleotide positions in the manuscript relate to M41 (GenBank accession number AY851295.1).

### *In vitro* growth curve.

Prior to infection CK cells seeded in six-well plates were washed once with phosphate buffered saline “a” (PBSa). CK cells were infected with 10^4^ PFU of rIBV or IBV and incubated for 1 h at either 37 or 41°C and 5% CO_2_; the inoculum was then removed. Cells were washed once with PBSa to remove unbound virus prior to the addition of 3 mL of BES [*N*,*N*-bis(2-hydroxyethyl)-2-aminoethanesulfonic acid] (73) medium per well. Cells were incubated at the same temperature as the first 1 h of incubation step. Supernatant from one well per time point was harvested at defined intervals postinfection (p.i.) and titrated in triplicate in CK cells to determine the quantity of infectious progeny. The experiment was performed three times in three different preparations of CK cells.

### Analysis of RNA synthesis by qRT-PCR.

Total RNA was extracted from harvested cell lysate by using RNeasy columns (Qiagen), including an on-column DNase digest. The total RNA (500 ng) was reverse transcribed using a random primer, 5′-GTTTCCCAGTCACGATCNNNNNNNNNNNNNNN-3′, and a Superscript IV reverse transcriptase kit (Invitrogen). The quantity of IBV-derived RNA was quantified using qRT-PCR with either primers targeting the N sgmRNA or primers targeting the 5′ UTR. Quantitative RT-PCR was performed using a TaqMan Universal Master Mix II, no UNG (Life Technologies, Waltham, MA), including 125 nM final probe and 500 nM final primers. Primers and probes have been published previously (74) and were used as follows: (i) for the detection of sgmRNA N, forward primer 5′-CTAGCCTTGCGCTAGATTTTTAACT-3′, reverse primer 5′-GAGAGGTACACGCGGGACAA-3′, and the N sgmRNA probe sequence 5′-FAM-ACAAAGCAGGACAAGCA-MGB-NFQ-3′ and (ii) for the detection of the 5′ UTR, forward primer 5′-GCTTTTGAGCCTAGCGTT-3′, reverse primer 5′-GCCATGTTGTCACTGTCTATTG-3′, and the probe 5-6-carboxyfluorescein (FAM)-CACCACCAGAACCTGTCACCTC-6-carboxytetramethylrhodamine (TAMRA)-3′. Thermocycling conditions included an initial hold at 95°C for 20 s, followed by 40 cycles of 95°C for 1 s, and then 60°C for 20 s. IBV RNA copy numbers were determined from standard curves generated from an 8-point 10-fold dilution series of plasmid containing the sequence amplified. The resulting threshold cycle (*C_T_*) results were used to calculate the log of relative RNA copies (log_10_) using the linear equation from the standard curve.

### Analysis of IFN-α and IFN-β expression by qRT-PCR.

A panel of reference genes was analyzed to select the most stable gene for appropriate samples normalization (75, 76). The panel included RPL13, HMBS, ACTB, HPRT1, B2M, and RPLPO, and the results were analyzed using geNorm (77) and Normfinder (78) to calculate stability values and to select the housekeeping gene fitting all the groups and conditions. Primers and probes specific for the assays targeting IFN-α, IFN-β, IL-1β, and IL-6 were designed by Primer Design. Briefly, quantitative PCR was performed on a 2-μL cDNA sample normalized to 50 ng/μL, 5 μL of TaqMan Fast Universal PCR 2× Master Mix (Applied Biosystems), and 3 μL of 300 nM final probe and 500 nM final primers and water to a final volume. Samples were run in 96-well plates using QuantStudio 5 with the default fast cycle conditions: 20 min at 95°C, 40 cycles of 1 s at 95°C, and then 20 s at a 60°C annealing temperature. qRT-PCR data were normalized using the housekeeping gene RPL13. ΔΔ*C_T_* analysis was performed using the relative quantification of gene expression, with an average Δ*C_T_* of the reference genes in comparison to each experimental gene. Samples were run in the same plate for the reference gene and the experimental gene at the same time to avoid interplate variations. Data were analyzed in GraphPad Prism using one-way analysis of variance (ANOVA). ANOVA data were corrected for multiple comparisons using the Bonferroni adjustment method. Differences between groups at that time point were considered significant at *P* < 0.05. The data are presented either as the log_10_-fold change or the fold change in relative mRNA gene expression of virus-infected versus mock-infected samples.

### Ethics statement.

Animal experimental protocols were carried out in accordance with the Home Office guidelines of the United Kingdom and under license granted for experiments involving regulated procedures on animals protected under the UK Animals (Scientific Procedures) Act 1986. Experiments were performed at the licensed experimental poultry house facilities at The Pirbright Institute (X24684464). All experiments were approved by the local animal welfare and ethical review committee. Chickens were provided by The National Avian Research Facility (The Roslin Institute).

### Homologous vaccine challenge *in vivo* experiment.

Groups of 30 SPF RIR chickens were raised in floor pens in separate positive-pressure, HEPA-filtered isolation rooms. At 8 days of age, each bird was inoculated with 0.1 mL of PBS for mock infection or 0.1 mL of PBS containing 10^4^ PFU of rIBV M41-R via the intraocular and intranasal route. At 27 days p.v., a challenge dose of either 0.1 mL of PBS for mock challenge or 0.1 mL of PBS containing 10^4^ PFU of M41-CK was also administered by the intraocular and intranasal routes. Birds were assessed both postvaccination (p.v.) and postchallenge (p.c.) for clinical signs, including snicking and the presence of tracheal rales, as previously described (26). Birds were culled by cervical dislocation at specific times p.i., and a panel of tissues, including tracheas, eyelids, and beaks, were sampled with sections stored in either RNAlater (Ambion, Thermo Fisher Scientific) or PBS for downstream analysis. Ciliary activity in harvested tracheas was assessed on day 4 p.v. and days 4 and 6 p.c., as previously described (37, 38). Blood samples were collected and processed for the collection of serum. Birds were deemed protected if 50% of ciliary activity was retained in 9 of 10 rings used for the assessment of ciliary activity (39).

### Assessment of the presence of IBV genomic material in harvested tissue samples.

Beak, eyelid, and trachea samples stored in RNAlater were homogenized using a Tissue Lyser II (27 Hz/s, 4 min). Homogenized samples were centrifuged to remove tissue debris, and 170 μL was used for RNA extraction with a Qiagen RNeasy minikit, according to the manufacturer’s instructions for RNA cleanup. RNA was reverse transcribed using a random primer (5′-GTTTCCCAGTCACGATCNNNNNNNNNNNNNNN-3′) and SuperScript IV according to the manufacturer’s (Invitrogen) protocol. The resulting cDNA was assessed by PCR using *Taq* DNA polymerase according to the manufacturer’s (Invitrogen) instructions and IBV-specific oligonucleotides targeting the 3′ UTR as previously described (79).

### Virus isolation from harvested tissue samples.

Trachea and eyelid samples, stored in PBS, harvested at days 2 and 4 p.v. and days 4 and 6 p.c., were homogenized as previously described (28). Ten-day-old embryonated eggs were inoculated with 100 μL of tissue derived supernatant via the allantoic cavity, at one egg per sample, and then incubated at 37°C for 24 h. Embryos were culled using a schedule 1 method, and the allantoic fluid was harvested, which was subsequently screened for viral presence by RT-PCR as described above.

### Assessment of IBV-specific serum antibody levels by ELISA.

Serum samples collected at day 14 p.v. (prechallenge), day 4 p.c., and day 14 p.c. were prepared from whole-blood samples from each bird by centrifugation at 3,000 rpm for 5 min. Sera were heat inactivated at 56°C for 30 min. Antibody levels were determined by ELISA using a commercial IBV ELISA kit (BioChek) for the detection of IBV-specific antibodies. Sera were diluted 1/80 in sample diluent buffer for analysis, and then the assay was performed according to the manufacturer’s instructions. All samples were run alongside BioChek reference control samples to confirm results. Each sample dilution was run in triplicate, and S/P ratios were calculated according to the following equation: (mean sample – mean negative control)/(mean positive control – mean negative control). S/P ratios greater than 0.2 were considered positive for IBV antibodies.

### Assessment of neutralizing antibody.

The quantity of M41-CK-specific neutralizing antibodies were assessed via plaque reduction assay in CK cells. Heat-inactivated serum samples, harvested 14 days p.c., were serially diluted (2-fold) in BES cell culture medium, starting at 1/5. Each serum dilution was incubated with 10^3^ PFU of M41-CK for 30 min at room temperature on an orbital shaker, and then the quantity of infectious virus in each sample was determined via titration in triplicate on CK cells. PRNT_50_ values were calculated according to the Reed-Muench method for endpoint titer determination.

### Statistics.

All statistical analyses were performed using GraphPad Prism version 8.0. Normality and the standard deviations for each data set were assessed prior to each statistical test.
